# 

**Published:** 1910-12-24

**Authors:** 


THE MODERN MEDICAL AND INSTITUTIONAL JOURNAL.

Telegraphic Address: Ospedale, London.

Telephone Number: 2734 Gerrard.

NEW SERIES. No. 203, Vol. VIII.

No. 1275, Vol. XLIX.

SATURDAY, DEC. 24TH, 1910.

PRICE THREEPENCE

